# Asundexian Versus Apixaban in Patients With Atrial Fibrillation

**DOI:** 10.1002/hsr2.71795

**Published:** 2026-03-16

**Authors:** Zara Saeed, Syeda Ariba Zehra, Sumyya Tariq, Rocio Pun Zumaran, Mounika Kotte, Zeeshan Hameed, Neeraj Joshi, Pushpa Kumari, Kanta Kumari, Awais Habib, Fawad Talat, Osama Taj, Jahanzeb Malik, Abida Perveen

**Affiliations:** ^1^ Department of Medicine Ibn e Seena Hospital Kabul Afghanistan; ^2^ Fauji Foundation Hospital Pakistan; ^3^ Universidad Autonoma de Guadalajara Mexico

**Keywords:** anticoagulation, asundexian, bleeding risk, Factor XIa inhibitor, thromboembolic prevention

## Abstract

**Background:**

Anticoagulation therapy is central to the prevention of thromboembolic disorders but is limited by bleeding complications associated with traditional agents such as warfarin and direct oral anticoagulants (DOACs). Factor XIa inhibition has emerged as a novel strategy aimed at reducing thrombotic risk while preserving hemostasis. Asundexian, a selective Factor XIa inhibitor, represents a promising advancement in this evolving therapeutic landscape.

**Methods:**

This narrative review synthesizes available preclinical and clinical evidence on Asundexian, with a focus on its pharmacological mechanism, efficacy, safety profile, and potential clinical applications. Key data from major clinical trials, including PACIFIC‐AF and PACIFIC‐STROKE, were reviewed to evaluate its role in contemporary anticoagulation therapy.

**Results:**

Clinical trial evidence indicates that Asundexian effectively reduces the incidence of stroke and systemic embolism in patients with atrial fibrillation and those at risk of recurrent ischemic stroke. Importantly, these benefits are achieved with a lower risk of major bleeding compared to conventional anticoagulants. Asundexian's fixed dosing regimen, oral administration, and lack of routine coagulation monitoring enhance treatment convenience and patient adherence. Emerging data also suggest potential applicability in other conditions requiring long‐term anticoagulation, such as venous thromboembolism and acute coronary syndrome.

**Conclusion:**

Asundexian offers a novel and potentially safer approach to anticoagulation by selectively targeting Factor XIa, balancing thromboembolic protection with reduced bleeding risk. While early clinical results are encouraging, further large‐scale and long‐term studies are required to establish its safety and efficacy across diverse patient populations. Asundexian has the potential to redefine standards of care in anticoagulation therapy and improve patient outcomes.

## 1 Introduction

Anticoagulation therapy is essential for managing and preventing thromboembolic disorders, including atrial fibrillation (AF), deep vein thrombosis (DVT), pulmonary embolism (PE), and stroke [1]. These conditions arise from abnormal blood clotting, which can obstruct blood vessels, leading to serious cardiovascular events or organ damage [2]. Anticoagulants work by inhibiting various coagulation cascade components, thereby reducing the risk of clot formation. Historically, warfarin and heparin were the mainstays of therapy, both requiring careful monitoring due to their narrow therapeutic windows and significant risk of bleeding [3].

The advent of direct oral anticoagulants (DOACs), such as rivaroxaban, apixaban, and dabigatran, represented a significant advancement in anticoagulation [4, 5]. These newer agents offered fixed dosing, fewer food and drug interactions, and no need for routine coagulation monitoring, making them more convenient for both patients and healthcare providers [6]. Despite these advancements, unmet needs in anticoagulation therapy persist. One of the most significant challenges remains the balance between preventing thromboembolic events and minimizing the risk of bleeding, particularly major bleeding events such as intracranial hemorrhage [7, 8].

While DOACs have a better safety profile compared to warfarin, the risk of bleeding, particularly in elderly patients or those with renal impairment, continues to be a major concern [9]. Additionally, for some patients, current anticoagulants are contraindicated due to their bleeding risk, leaving them without effective preventive options for conditions like AF [10]. Furthermore, certain patient populations, such as those with mechanical heart valves or severe kidney disease, may not be suitable for DOACs, highlighting the need for new therapies that can provide effective anticoagulation with fewer adverse effects [11].

In response to these challenges, researchers have turned to novel targets in the coagulation cascade, aiming to develop agents that retain efficacy while minimizing bleeding risks. Factor XI, a key component in the intrinsic coagulation pathway, has emerged as a promising target [12]. Inhibition of Factor XI reduces the risk of pathological clot formation without significantly impairing hemostasis, thereby lowering the bleeding risk [13]. This approach represents a new direction in anticoagulation therapy, potentially addressing some of the most critical unmet needs in the field. Asundexian, a novel Factor XIa inhibitor, is at the forefront of this development, offering the potential for a more precise and safer approach to anticoagulation [14, 15, 16].

## 2 The Development of Factor XIa Inhibitors

The development of Factor XIa inhibitors represents a significant shift in the approach to anticoagulation therapy. Historically, most anticoagulants have targeted various components of the coagulation cascade, such as Factor Xa or thrombin, both of which play critical roles in the formation of blood clots [17]. While these agents have been effective at reducing the incidence of thromboembolic events, they are also associated with an increased risk of bleeding, particularly major bleeding episodes that can have severe consequences for patients [18]. The search for safer anticoagulants has led researchers to focus on Factor XI, a component of the intrinsic pathway of the coagulation cascade, which has shown potential for selective inhibition of clot formation without significantly compromising normal hemostasis [19].

Factor XI was first identified as a promising target due to its role in the amplification phase of clot formation [20]. Unlike factors in the extrinsic pathway, which are crucial for the rapid initiation of clotting in response to vascular injury, Factor XI plays a more secondary role, enhancing thrombin generation during sustained coagulation. This led to the hypothesis that inhibiting Factor XI could reduce the risk of thrombosis without greatly increasing the likelihood of spontaneous bleeding. Clinical and epidemiological observations have supported this theory. Individuals with congenital Factor XI deficiency (a condition known as hemophilia C) tend to experience fewer thromboembolic events and, notably, have a much lower incidence of spontaneous bleeding compared to individuals with deficiencies in other clotting factors [21]. These insights laid the groundwork for developing agents that could selectively inhibit Factor XI activity as a novel therapeutic strategy.

The review by Cortese et al. [22] highlights the challenges of anticoagulation in patients with AF and renal impairment, emphasizing that while apixaban offers improved safety and efficacy compared to warfarin, bleeding risk remains a significant concern in this high‐risk population. These findings underscore the unmet clinical need for anticoagulants that can provide effective thromboembolic protection while further minimizing bleeding complications. Factor XIa inhibitors, such as asundexian, directly address this gap by selectively targeting the intrinsic coagulation pathway, theoretically reducing pathological clot formation without substantially impairing hemostasis. Consequently, the work of Cortese and colleagues supports the rationale for the development of asundexian and similar agents, particularly for patient populations, including those with renal dysfunction, who are vulnerable to bleeding with current therapies, highlighting the potential for Factor XIa inhibition to offer safer and more effective anticoagulation in clinical practice [22].

Fioretti et al. [23] review recent advances in anticoagulation therapy, emphasizing the need for safer agents that reduce thromboembolic risk while minimizing bleeding, particularly in high‐risk cardiovascular populations. The authors highlight the limitations of current therapies, including DOACs and warfarin, noting that bleeding complications remain a major clinical concern. This review supports the rationale for developing Factor XIa inhibitors such as asundexian, which selectively target the intrinsic coagulation pathway to prevent pathological clot formation while preserving normal hemostasis. Incorporating the insights from Fioretti and colleagues reinforces the potential of asundexian to provide effective and safer anticoagulation across diverse patient populations, including those at elevated bleeding risk [23].

The preclinical development of Factor XIa inhibitors involved a series of studies aimed at confirming the anticoagulant potential of targeting this factor. Animal models, particularly those involving thrombosis, demonstrated that inhibiting Factor XI effectively reduced clot formation without significantly increasing bleeding tendencies [24]. These early studies validated the approach and supported the initiation of clinical trials in humans.

Asundexian, a small‐molecule Factor XIa inhibitor, has emerged as a leading candidate in this class of anticoagulants. Preclinical studies demonstrated its ability to selectively inhibit Factor XIa, reducing thrombin generation and platelet aggregation while maintaining a favorable safety profile [25]. Initial Phase 1 clinical trials confirmed these findings, showing that asundexian effectively suppressed Factor XIa activity without causing a marked increase in bleeding, even at therapeutic doses [26]. Following these early successes, larger Phase 2 studies have been initiated to assess its efficacy and safety in preventing thromboembolic events in patients at high risk, such as those with AF or recent stroke [27]. These studies are part of a broader effort to explore the therapeutic potential of Factor XIa inhibitors in various clinical settings (Table 1).

**Table 1:** Factor XI/XIa inhibitors with concluded/undergoing Phase II clinical studies.

Drug name	Type of agent	Mechanism of action	Administration frequency	Concluded studies	Comparator	Phase	Number of patients	Study population
Asundexian	Small molecule	Inhibits Factor XIa	Once daily	PACIFIC‐AF—NCT04218266	Apixaban	II	755	Atrial fibrillation
				PACIFIC‐STROKE—NCT04304508	Placebo	II	1808	Noncardioembolic stroke
				PACIFIC‐AMI—NCT04304534	Placebo	II	1601	After acute myocardial infarction
Milvexian	Small molecule	Inhibits Factor XIa	Once/twice daily	AXIOMATIC‐SSP—NCT03766581	Placebo	II	2295	Noncardioembolic stroke
				AXIOMATIC‐TKR—NCT03891524	Enoxaparin	II	1242	Major orthopedic surgery
				NCT03000673	Enoxaparin/unfractionated heparin	II	32	End‐stage renal disease
SHR2285	Small molecule	Inhibits Factor XIa	Twice daily	NCT05203705	Enoxaparin	II	500	Major orthopedic surgery
Abelacimab	Human monoclonal antibody	Binds to Factor XI and prevents its activation by Factor XIIa or thrombin	Once a month	ANT‐004—NCT04213807	Placebo	II	28	Atrial fibrillation
				AZALEA‐TIMI 71—NCT04755283	Rivaroxaban	II	1287	Atrial fibrillation
				ANT‐005 TKA—EudraCT number: 2019–003756‐37	Enoxaparin	II	412	Major orthopedic surgery
Fesomersen	Antisense oligonucleotide	Binds to Factor XI mRNA, blocking its translation	Once a month	RE‐THINc ESRD—NCT04534114	Placebo	II	307	End‐stage renal disease
IONIS‐FXIRX/FXI‐ASO	Antisense oligonucleotide	Binds to Factor XI mRNA, blocking its translation	Once a week	FXI‐ASO TKA—NCT01713361	Enoxaparin	II	300	Major orthopedic surgery
				NCT02553889	Placebo	II	49	End‐stage renal disease
				EMERALD—NCT03358030	Placebo	II	213	End‐stage renal disease
Osocimab	Human monoclonal antibody	Inhibits Factor XIa, preventing activation of Factor IX	Once a month	FOXTROT—NCT03276143	Apixaban/Enoxaparin	II	813	Major orthopedic surgery
				CONVERT—NCT04523220	Placebo	II	704	End‐stage renal disease
Xisomab 3G3	Recombinant humanized monoclonal antibody	Binds to Factor XI, preventing its activation	—	NCT03612856	Placebo	II	27	End‐stage renal disease

### 2.1 Pharmacology and Mechanism of Action of Asundexian

Asundexian is a novel anticoagulant that works by selectively inhibiting Factor XIa, an enzyme crucial to the amplification phase of the coagulation cascade [25, 26]. Unlike other anticoagulants that target more central components of the clotting process, such as Factor Xa or thrombin, asundexian's selective inhibition of Factor XIa offers a unique balance between efficacy and safety. This selective inhibition minimizes the risk of pathologic clot formation while preserving normal hemostasis, thereby reducing the likelihood of spontaneous bleeding—a major concern with conventional anticoagulants [19, 28].

**Figure 1:**
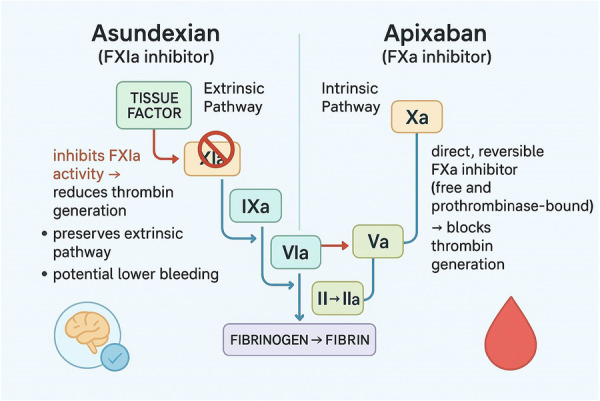
Mechanistic comparison of asundexian (FXIa inhibitor) and apixaban (FXa inhibitor) in the coagulation cascade.

Mechanistically, Factor XIa plays a key role in the intrinsic pathway of coagulation, which is primarily activated by the sustained activation of thrombin and the stabilization of clots [20]. When vascular injury occurs, the extrinsic pathway is rapidly activated to initiate clot formation, while the intrinsic pathway, where Factor XI operates, further amplifies and maintains clot stability [29]. By inhibiting Factor XIa, asundexian interferes with the amplification phase of thrombin generation, thus reducing excessive clot formation without significantly impairing the body's ability to respond to vascular injury through the extrinsic pathway. This allows the agent to reduce thromboembolic events, such as those seen in AF or venous thromboembolism (VTE), without greatly increasing the risk of bleeding (Figure 1) [27, 30].

Compared to DOACs such as rivaroxaban or apixaban, which target Factor Xa, asundexian's inhibition of Factor XIa offers several potential advantages. Factor Xa inhibitors are highly effective at preventing clot formation but are also associated with a substantial bleeding risk [31]. Asundexian, by contrast, targets a step further downstream in the coagulation cascade, interfering with clot formation at a stage where thrombin amplification is taking place [26, 32].

Additionally, compared to older anticoagulants like warfarin, which requires frequent monitoring and dosage adjustments due to its narrow therapeutic window and numerous drug and food interactions, asundexian offers a more convenient option [3, 27]. It does not require routine coagulation monitoring, nor does it interact with many foods or medications, similar to the advantage that DOACs brought over warfarin [4, 5, 6]. However, asundexian further improves on this by offering a potentially superior safety profile due to its reduced bleeding risk [25, 28].

When compared to other novel agents under development, such as monoclonal antibodies or antisense oligonucleotides targeting Factor XI, asundexian stands out due to its oral administration and rapid reversibility [33]. Monoclonal antibodies like osocimab or antisense oligonucleotides like IONIS‐FXI target Factor XI or its precursors, but they often require subcutaneous or intravenous administration, which limits their practicality for long‐term use in ambulatory patients [34, 35]. Asundexian's oral formulation offers a significant advantage in terms of patient compliance and ease of use in a chronic setting (Table 1) [26, 27].

### 2.2 Clinical Trial Evidence for Asundexian

The clinical trial program for asundexian has been focused on assessing its efficacy and safety in preventing thromboembolic events in high‐risk patient populations. Key trials in this program include PACIFIC‐AF and PACIFIC‐STROKE, which have provided valuable data (Table 2) [27, 36].

**Table 2:** Randomized clinical trials of factor XI/XIa inhibitor in comparison to placebo/alternative therapies, categorized on the basis of the study setting.

Study	Active drug	Comparator	Phase of study	Current stage
*Atrial Fibrillation*				
LIBREXIA‐AF—NCT05757869	Milvexian	Apixaban	III	Ongoing
PACIFIC‐AF—NCT04218266	Asundexian	Apixaban	II	Finished
OCEANIC‐AF—NCT05643573	Asundexian	Apixaban	III	Stopped
ANT‐004—NCT04213807	Abelacimab	Placebo	II	Finished
AZALEA‐TIMI 71—NCT04755283	Abelacimab	Rivaroxaban	II	Finished
LILAC‐TIMI 76—NCT05712200	Abelacimab	Placebo	III	Ongoing
*Noncardioembolic Stroke*				
AXIOMATIC‐SSP—NCT03766581	Milvexian	Placebo	II	Finished
LIBREXIA‐STROKE—NCT05702034	Milvexian	Placebo	III	Ongoing
PACIFIC‐STROKE—NCT04304508	Asundexian	Placebo	II	Finished
OCEANIC‐STROKE—NCT05686070	Asundexian	Placebo	III	Ongoing
*After Myocardial Infarction*				
LIBREXIA‐ACS—NCT05754957	Milvexian	Placebo	III	Ongoing
PACIFIC‐AMI—NCT04304534	Asundexian	Placebo	II	Finished
*Venous Thromboembolism Prevention in Cancer Patients*				
ASTER—NCT05171049	Abelacimab	Apixaban	III	Ongoing
MAGNOLIA—NCT05171075	Abelacimab	Dalteparin	III	Ongoing
*Major Orthopedic Surgery*				
AXIOMATIC‐TKR—NCT03891524	Milvexian	Enoxaparin	II	Finished
FOXTROT—NCT03276143	Osocimab	Apixaban/Enoxaparin	II	Finished
FXI‐ASO TKA—NCT01713361	FXI‐ASO (ISIS 416858)	Enoxaparin	II	Finished
ANT‐005 TKA—EudraCT number: 2019‐003756‐37	Abelacimab	Enoxaparin	II	Finished
NCT05203705	SHR2285	Enoxaparin	II	Ongoing
*Chronic Kidney Disease and End‐Stage Renal Disease*				
NCT03000673	Milvexian	Unfractionated heparin/Enoxaparin	II	Finished
NCT03787368	Osocimab	Placebo	I	Finished
CONVERT—NCT04523220	Osocimab	Placebo	II	Finished
NCT02553889	FXI‐ASO (ISIS 416858)	Placebo	II	Finished
EMERALD—NCT03358030	FXI‐ASO (ISIS 416858)	Placebo	II	Finished
RE‐THINc ESRD—NCT04534114	Fesomersen	Placebo	II	Finished
NCT03612856	Xisomab 3G3	Placebo	II	Finished
MK‐2060‐004—NCT03873038	MK‐2060	Placebo	I	Finished
MK‐2060‐007—NCT05027074	MK‐2060	Placebo	II	Ongoing
MK‐2060‐011—NCT05656040	MK‐2060	Placebo	I	Ongoing
MK‐2060‐012—NCT05769595	MK‐2060	Placebo	I	Ongoing

The PACIFIC‐AF trial evaluated efficacy in AF. It was a randomized, double‐blind, dose‐finding study comparing asundexian to apixaban [36]. Results showed that asundexian demonstrated noninferior efficacy compared to apixaban in preventing stroke/systemic embolism, with significantly lower rates of major bleeding, including intracranial and gastrointestinal bleeding [36].

The PACIFIC‐STROKE trial investigated asundexian in patients with recent noncardioembolic ischemic stroke [37]. Asundexian reduced recurrent ischemic stroke incidence compared to placebo and showed lower rates of major bleeding than conventional anticoagulants [37, 38].

Across ongoing trials (including VTE and ACS populations), asundexian has consistently demonstrated favorable efficacy and a superior bleeding profile compared to standard anticoagulants [27, 36, 37, 38].

In terms of tolerability, asundexian has shown mild adverse events (GI discomfort, headache, dizziness) without major liver toxicity or drug–drug interactions [26, 27]. This further strengthens its potential role as a next‐generation oral anticoagulant.

## 3 Advantages of Asundexian Over Current Anticoagulants

Asundexian offers several key advantages over current anticoagulants, with reduced bleeding risk being its most significant differentiator [39, 40]. One of the primary challenges with existing anticoagulants, such as warfarin and DOACs, is the increased risk of bleeding, particularly in high‐risk areas like the brain (intracranial hemorrhage) and gastrointestinal tract [41]. Asundexian, through its selective inhibition of Factor XIa, effectively reduces clot formation without entirely suppressing thrombin generation, which is essential for normal clotting processes [42, 43]. This unique mechanism allows asundexian to lower the likelihood of pathological clot formation while maintaining enough hemostatic function to minimize the risk of bleeding, particularly major and life‐threatening bleeds [44]. In clinical trials, asundexian has consistently shown a lower rate of bleeding complications compared to other anticoagulants, making it an attractive option for patients who require long‐term anticoagulation but are at heightened risk of bleeding [45, 46].

Another advantage of asundexian is its straightforward dosing regimen and favorable pharmacokinetics, which support better patient adherence [47]. Unlike warfarin, which requires frequent monitoring and dose adjustments due to its narrow therapeutic window and sensitivity to dietary changes, asundexian offers fixed dosing without the need for routine coagulation monitoring [48]. This simplifies the management of anticoagulation therapy, reducing the burden on both patients and healthcare providers. Moreover, asundexian does not have significant interactions with foods or other medications, which is a common concern with warfarin [49]. This increases its convenience and safety, particularly for patients on multiple medications [50].

Asundexian's pharmacokinetic profile also supports patient adherence. Its oral administration makes it easier for patients to take regularly, and its long half‐life allows for once‐daily dosing, ensuring steady drug levels and reducing the risk of missed doses [51]. In comparison to anticoagulants that require more frequent dosing or injections (such as low‐molecular‐weight heparins), asundexian's dosing regimen is simpler, encouraging better adherence and long‐term effectiveness in preventing thromboembolic events [52].

### 3.1 Place‐in‐Therapy of Asundexian

Asundexian represents a novel addition to the anticoagulation armamentarium, complementing existing DOACs such as apixaban, rivaroxaban, and dabigatran. Unlike conventional DOACs, which target central components of the coagulation cascade like Factor Xa or thrombin, asundexian selectively inhibits Factor XIa, modulating the intrinsic pathway to reduce thrombus formation while preserving normal hemostasis [22]. This targeted mechanism offers a potentially safer option for patients at high risk of bleeding, including the elderly, those with renal impairment, or patients requiring long‐term anticoagulation. While DOACs remain first‐line agents for most indications such as nonvalvular AF and VTE, asundexian may occupy a niche in populations where bleeding risk is a critical limitation of standard therapy. Furthermore, its oral formulation, fixed dosing, and favorable pharmacokinetic profile make it a convenient alternative in both outpatient and chronic care settings. Ongoing clinical trials will help define its precise role, potentially positioning asundexian as either a primary option in high‐bleeding‐risk patients or as an adjunctive therapy in combination with other antithrombotic strategies [53]. Table 4 shows key differences and similarities between asundexian, a Factor XIa inhibitor, and conventional DOACs, highlighting mechanism, bleeding risk, dosing, and potential clinical applications.

### 3.2 Potential Clinical Applications of Asundexian

Asundexian has the potential for wide‐ranging clinical applications, especially in conditions where long‐term anticoagulation is essential. One of its primary uses is in AF, a common arrhythmia that significantly increases the risk of stroke. For patients with AF, preventing stroke and other thromboembolic events is critical, and anticoagulation is the cornerstone of treatment [53]. While current anticoagulants, like warfarin and DOACs, are effective, they come with an increased risk of bleeding [54]. Asundexian, with its ability to reduce clot formation without heavily impairing normal clotting, offers a safer alternative, potentially allowing AF patients to maintain protection against stroke with lower bleeding risk [55].

Stroke prevention is another important application for asundexian, particularly in patients who have already experienced a noncardioembolic ischemic stroke [56]. These individuals are at high risk for recurrent strokes, and while antiplatelet therapy is commonly used, adding anticoagulation is often necessary for more robust protection [57]. Asundexian, due to its reduced bleeding risk compared to traditional anticoagulants, may offer a safer option for these patients, providing effective stroke prevention without the high risk of bleeding complications that can be seen with standard treatments [58].

Asundexian could also be beneficial in the prevention and treatment of VTE, which includes DVT and PE [59]. These conditions require anticoagulation to prevent clot extension and recurrence, but the challenge again lies in balancing the reduction of thromboembolism risk with minimizing the danger of bleeding [60]. Asundexian's targeted mechanism of action makes it a promising candidate for managing VTE, offering an effective treatment option with potentially fewer bleeding risks compared to DOACs [61].

### 3.3 Challenges and Limitations of Asundexian

Despite its potential, asundexian also faces several challenges and limitations that need to be carefully considered [62]. One of the major concerns is the lack of long‐term data on its safety and efficacy across diverse patient populations [63]. While clinical trials so far have demonstrated promising results, larger and longer studies are required to confirm whether asundexian can consistently provide stroke prevention and VTE management with reduced bleeding risk compared to established anticoagulants [64]. Additionally, the trials conducted to date have largely been phase II studies, meaning that definitive Phase III data are still pending [65].

Another limitation is that while asundexian reduces bleeding risk, it may not be as effective in preventing thrombosis in certain high‐risk patient groups, such as those with mechanical heart valves or advanced thrombophilia [66]. Traditional anticoagulants like warfarin are still the standard of care for such patients, and it remains uncertain whether asundexian can provide adequate protection in these complex cases [67]. Furthermore, the high cost of novel anticoagulants could limit accessibility, particularly in low‐ and middle‐income countries where warfarin remains widely used due to its affordability [68].

### 3.4 Future Directions

Looking ahead, ongoing Phase III clinical trials will be crucial to establishing asundexian's role in the anticoagulation landscape [69]. Trials such as the PACIFIC‐AF, PACIFIC‐STROKE, and PACIFIC‐AMI programs are expected to provide further insights into its safety, efficacy, and potential advantages over existing therapies [70]. If the results confirm early findings, asundexian could become a first‐line therapy for patients requiring anticoagulation, particularly those at high risk of bleeding (Table 3) [71].

**Table 3:** Ongoing Phase III RCTs of Factor XI/XIa inhibitor in comparison to placebo/alternative therapies.

Study	Number of patients	Active drug	Dosage	Comparator	Dosage
*Atrial Fibrillation*					
LIBREXIA‐AF—NCT05757869	15,500	Milvexian	100 mg twice daily	Apixaban	2.5/5 mg twice a day
LILAC‐TIMI 76—NCT05712200	1900	Abelacimab	150 mg subcutaneously once a month	Placebo	—
Noncardioembolic Stroke					
LIBREXIA‐STROKE—NCT05702034	15,000	Milvexian	Once or twice daily	Placebo	—
OCEANIC‐STROKE—NCT05686070	9300	Asundexian	Once daily	Placebo	—
*After Myocardial Infarction*					
LIBREXIA‐ACS—NCT05754957	16,000	Milvexian	Once or twice daily	Placebo	—
*VTE Prevention in Cancer Patients*					
ASTER—NCT05171049	1655	Abelacimab	150 mg intravenously followed by 150 mg subcutaneously monthly	Apixaban	10 mg followed by 5 mg twice a day
MAGNOLIA—NCT05171075	1020	Abelacimab	150 mg intravenously followed by 150 mg subcutaneously monthly	Dalteparin	200 IU/kg/day followed by 150 IU/kg/day

**Table 4:** Comparison of asundexian and conventional direct oral anticoagulants (DOACs).

Feature	Asundexian	Conventional DOACs (apixaban, rivaroxaban, dabigatran)	Clinical implications
Target	Factor XIa (intrinsic pathway)	Factor Xa or thrombin (central coagulation pathway)	Selective targeting may reduce bleeding risk
Mechanism of action	Inhibits amplification of thrombin generation without significantly affecting normal hemostasis	Inhibits key coagulation steps, strongly reducing thrombin generation	Preserves physiological clotting more than DOACs
Bleeding risk	Lower, particularly major bleeding (intracranial, gastrointestinal)	Moderate to high; risk of major bleeding events persists	Advantage in high‐risk populations (elderly, renal impairment)
Renal considerations	Dose adjustment required in severe impairment; favorable profile in mild‐to‐moderate CKD	Dose adjustment required; some agents heavily renally cleared (e.g., dabigatran)	May offer safer anticoagulation in CKD patients
Dosing	Oral, fixed, once daily	Oral, fixed, 1–2 times daily depending on drug	Simplifies adherence; comparable convenience to DOACs
Monitoring	Not required	Not required	Both avoid routine coagulation monitoring needed for warfarin
Drug–food interactions	Minimal	Minimal for most DOACs; some may require food considerations (e.g., rivaroxaban)	More convenient than warfarin
Reversibility	Rapid onset/offset; specific reversal strategies under development	Rapid offset; reversal agents available (idarucizumab for dabigatran, andexanet alfa for Xa inhibitors)	Reversal options exist, but asundexian strategies still in development
Clinical indications (under investigation)	Atrial fibrillation, venous thromboembolism, stroke prevention, acute coronary syndrome	Atrial fibrillation, venous thromboembolism, stroke prevention, acute coronary syndrome	Asundexian may initially be used in patients at high risk of bleeding
Formulation	Oral small molecule	Oral (dabigatran, apixaban, rivaroxaban)	Oral route supports outpatient and chronic use

In addition, research may explore combination strategies where asundexian is paired with antiplatelet therapies to optimize outcomes in patients with cardiovascular disease [72]. There is also growing interest in the use of precision medicine to identify which patients are most likely to benefit from Factor XIa inhibitors based on genetic, clinical, and biomarker profiles [73]. These strategies could maximize efficacy while minimizing risks, making asundexian a cornerstone of individualized anticoagulation therapy in the future [74].

## 4 Conclusion

Asundexian represents an exciting advancement in the field of anticoagulation, offering a promising alternative to traditional agents such as warfarin and DOACs [75]. By selectively inhibiting Factor XIa, it reduces thrombotic risk while preserving much of the body's natural ability to clot, thereby minimizing bleeding complications [76]. Its simple dosing regimen, lack of routine monitoring, and fewer food or drug interactions further enhance its clinical appeal [77].

Although challenges remain, including the need for long‐term data, cost considerations, and establishing efficacy in high‐risk populations, asundexian's potential to shift the balance between thrombosis prevention and bleeding risk is significant [78]. With ongoing clinical trials expected to provide more definitive evidence, asundexian may soon emerge as a safer and more effective anticoagulant, shaping the future of thromboembolic disease management [79].

## Author Contributions

**Zara Saeed:** conceptualization, methodology, investigation, writing – original draft. **Syeda Ariba Zehra:** conceptualization, methodology, validation, visualization, writing – review & editing. **Sumyya Tariq:** conceptualization, validation, investigation. **Rocio Pun Zumaran:** writing – original draft, methodology, data curation. **Mounika Kotte:** writing – original draft, supervision, resources, writing – review & editing. **Zeeshan Hameed:** conceptualization, writing – original draft, project administration. **Neeraj Joshi:** conceptualization, software, writing – review & editing. **Pushpa Kumari:** methodology, project administration, writing – review & editing. **Kanta Kumari:** methodology, validation, writing ‐ review & editing. **Awais Habib:** software, formal analysis, project administration, writing ‐ review & editing. **Fawad Talat:** data curation, supervision, resources, writing – review & editing. **Osama Taj:** methodology, project administration, writing – review & editing. **Jahanzeb Malik:** methodology, validation, writing – review & editing. **Abida Perveen:** methodology, software, project administration, writing – review & editing.

## Funding

The authors received no specific funding for this work.

## Disclosure

The lead/corresponding author (Abida Perveen) affirms that this manuscript is an honest, accurate, and transparent account of the study being reported; that no important aspects of the study have been omitted; and that any discrepancies from the study as planned (and, if relevant, registered) have been explained.

## Ethics Statement

The authors have nothing to report.

## Conflicts of Interest

The authors declare no conflicts of interest.

## Data Availability

Data sharing is not applicable to this article as no new data were created or analyzed in this study.
